# Comparison of Mobile Phone and CCD Cameras for Electrochemiluminescent Detection of Biogenic Amines

**DOI:** 10.3390/s22187008

**Published:** 2022-09-16

**Authors:** Nic Heckenlaible, Sarah Snyder, Patrick Herchenbach, Alyssa Kava, Charles S. Henry, Erin M. Gross

**Affiliations:** 1 Department of Chemistry and Biochemistry, Creighton University, Omaha, NE 68178, USA; 2 Department of Chemistry, Colorado State University, Fort Collins, CO 80523, USA

**Keywords:** electrochemiluminescence, carbon electrode, Ru(bpy)_3_^2+^, mobile phone detection

## Abstract

Biogenic amines are an important and widely studied class of molecules due to their link to the physiological processes of food-related illnesses and histamine poisoning. Electrochemiluminescent (ECL) detection offers an inexpensive and portable analytical method of detection for biogenic amines when coupled with recent advancements in low-cost carbon-based electrodes and a smartphone camera. In this work, a mobile phone camera was evaluated against a piece of conventional instrumentation, the charge-coupled device, for the detection of ECL from the reaction of biogenic amines with the luminescent compound tris(2,2′-bipyridyl)ruthenium(II). Assisted by a 3D-printed light-tight housing, the mobile phone achieved limits of detection of 127, 425 and 421 μM for spermidine, putrescine, and histamine, respectively. The mobile phone’s analytical figures of merit were lesser than the CCD camera but were still within the range to detect contamination. In an exploration of real-world samples, the mobile phone was able to determine the contents of amines in skim milk on par with that of a CCD camera.

## 1. Introduction

Biogenic amines (BA) are thermostable and non-volatile low molecular weight nitrogenous organic bases [1,2,3,4,5,6,7,8,9,10]. They are interesting from an analytical chemistry perspective as their presence at certain levels can be an indicator of poor food quality. They can serve as a marker for microbial contamination as they are formed as a result of microbial decarboxylation of amino acids by microorganisms. They can arise from contamination during processing, indicating poor hygiene in food-processing facilities. BA not only affect the flavor and aroma of foods but can make people sick. Histamine (Figure S1), in particular, has a high toxicity. To assess food quality quickly, it is important to have fast, portable, and inexpensive analytical methods that can measure individual BA in food samples, but also provide information about a total BA level in the food. BA can be tricky to conveniently detect and measure in this manner [11,12,13,14,15,16,17,18,19,20,21,22]. Many of these species, particularly polyamines such as spermidine and putrescine (Figure S1), lack a chromophore required for HPLC-UV detection, and would require derivatization [15]. BA also are not volatile and again require derivatization for gas chromatography or mass spectrometry detection [16]. Electrochemical methods offer speed, low cost, and small size. However, direct amine oxidation can be difficult and BA are well known to foul electrode surfaces. Pulsed-amperometric detection methods [18,19,20] and diamond electrodes [21] have successfully been applied to BA detection but are more complex and costly compared to conventional electrochemical methods. An ideal method for BA detection in food samples would be simple, portable, fast, and inexpensive for ease of implementation in food processing and storage facilities.

The electrochemical method, electrogenerated chemiluminescence (ECL, or electrochemiluminescence), has been coupled with capillary electrophoresis (CE) [23,24,25,26,27] and used on its own [28,29] to detect various biogenic amines. ECL [30,31,32,33,34,35,36] combines the qualities of electrochemical methods (inexpensive, easy to miniaturize) with the sensitivity of luminescent measurements. ECL also provides some inherent selectivity to an analytical method, as the species being detected both must oxidize under the potential applied and participate in the ECL reaction with the luminescent species. BA have been shown to participate in the oxidative-reductive ECL reaction with tris(2,2′-bipyridyl)ruthenium(II) (Ru(bpy)_3_^2+^, Figure S1) [23,24,25,26,27,28,29]. An ECL mechanism between Ru(bpy)_3_^2+^ and amine-containing species is shown in Figure S2 [37,38]. At a sufficient potential, Ru(bpy)_3_^2+^ is heterogeneously oxidized at the electrode (Step 1). The amine co-reactant must also be oxidized: either homogeneously by the electrogenerated Ru(bpy)_3_^3+^ (Step 2a) or at the electrode (Step 2b). The oxidized amine is not stable and rearranges into a free radical species (Step 3) which reduces the Ru(bpy)_3_^3+^, producing a photon with emission centered around 620 nm (Steps 4–5). In our detection method, the Ru(bpy)_3_^2+^ concentration is in excess, and the concentration of the co-reactant species can be measured as the ECL signal is proportional to its concentration. In this case, the amine should primarily be oxidized homogeneously, as shown in step 2a [37].

ECL detection can be made more accessible by the use of inexpensive and robust disposable electrodes rather than the more costly and maintenance-heavy precious metal electrodes commonly used. Paper-based electrodes have found success in ECL analysis [39,40,41] and more widely in the consumer market as the basis for disposable test strips. A method of stencil-printing carbon electrodes onto transparencies has been demonstrated as a way of inexpensively and efficiently fabricating electrodes for electrochemical analysis, such as anodic stripping voltammetry [42,43,44]. The work in this study extends the electrochemical applications to ECL analysis. The other modification that could be made in the name of accessibility is to the optical detector component of ECL instrumentation. Photomultiplier tubes (PMTs) and charge-coupled devices (CCDs) are capable of sensitive measurements of light intensity and are usually selected for ECL studies because chemiluminescent signals are low. These instruments are restricted to in-lab usage by their cost and requirements for a dark room. Mobile phones, on the other hand, are a nearly ubiquitous handheld tool and possess increasingly sensitive optical sensors with their built-in cameras. Mobile phones have been shown to be a viable detector for ECL analysis and have been applied to the detection of 2-(dibutylamino)ethanol, nicotinamide adenine dinucleotide (NADH), and proline [45,46]. In this work, we fabricate stencil-printed carbon electrodes on transparencies to generate ECL from the reaction of biogenic amines and Ru(bpy)_3_^2+^ and use a mobile phone to detect and quantitate the ECL signal. We then compare the use of a mobile phone detector with a CCD camera detector. As of yet, the ECL detection of biogenic amines has not been evaluated with this method. We use the ECL reaction between DBAE and Ru(bpy)_3_^2+^ to optimize the detection system with a 3D-printed light-tight box. Then, we apply the method to the detection of biogenic amines. This work demonstrates the possibility of a portable and low-cost ECL analysis system for use outside of a conventional laboratory setting. It shows how a robust ECL detection system based around a mobile phone camera can make analytical and safety-relevant measurements of ECL intensity to determine biogenic amine concentrations in real-world samples.

## 2. Materials and Methods

*Materials and Reagents.* Tris(2,2′-bipyridyl)dichlororuthenium(II) hexahydrate was obtained from Strem Chemicals. Biogenic amine (spermidine, putrescine, histamine) hydrochloride salts, 2-(dibutylamino)ethanol (99%), D-(+)-glucose, β-lactose, L-glutamic acid, casein, and graphite powder (<20 μm, synthetic) were obtained from Sigma-Aldrich (St. Louis, MO, USA). Monosodium phosphate monohydrate, boric acid, calcium chloride dihydrate, magnesium chloride hexahydrate, ferric chloride hexahydrate, cupric sulfate pentahydrate, L-lysine, and L-alanine were obtained from Fisher Scientific (Waltham, MA USA). Poly(dimethylsiloxane) silver paint (High Purity) was obtained from Structure Probe, Inc. (West Chester, PA, USA). Carbon ink (E3178) was obtained from Ercon (Wareham, MA, USA). All chemicals were reagent grade or higher. Transparency film (polyethylene terephthalate/PET) and double-sided adhesive (467 MP) were purchased from 3 M (Minneapolis, MN, USA). The electrode stencil and solution reservoir were designed using CorelDRAW (Corel, ON, Canada) and cut using a 30 W Epilog Engraver Zing CO_2_ laser cutter and engraver (Golden, CO, USA). Samples of Hiland Skim Milk were obtained from local retailers in the Omaha, Nebraska area. Solutions throughout were prepared using a 0.10 M pH 9.00 boric acid buffer unless otherwise stated.

*Fabrication of Stencil-Printed Carbon Electrodes*. A similar fabrication of stencil-printed carbon electrodes (SPCE) has previously been described in the literature for electrochemical applications [42]. A 3:5 ratio (w/w) of graphite powder to carbon ink was mixed thoroughly to form a paste that was then stencil-printed through a laser-cut stencil onto standard printer-size transparency sheets. The stencil formed the carbon paste into a pattern with three distinct electrodes. Figure S3A,B show this procedure using a rubber squeegee to apply the carbon paste. The electrode pattern was allowed to dry at room temperature before a coating of SPI Supplies conductive silver paint was applied to the terminus of the reference lead. The ink typically dries within 30 min. Lastly, a small laser-cut ring of packing tape (inner diameter 1 cm) was placed around the working surface of the electrode to act as a fluid reservoir. A SPCE with potentiostat leads attached is shown in Figure S3C.

*Electrode Characterization and ECL Generation and Collection*. All electrochemical experiments were carried out using a CH Instruments 600E series electrochemical analyzer (Austin, TX, USA). Unless otherwise noted, Ag ink served as the reference electrode, and screen-printed carbon served as the counter (Figure S3C). For ECL experiments, optical detection was performed either by a Samsung Galaxy S7 Edge mobile phone or by a ThorLabs DCU224M Charge-Coupled Device affixed with a MVL6WA 6 mm EFL lens. The Samsung Galaxy S7 Edge was operated in “Pro” mode with an exposure time of 10 s, a neutral white balance setting (5500 K), and a variable ISO setting (50–800). The ISO setting was adjusted for each analyte to maximize sensitivity while preventing saturation. The ThorLabs CCD was also operated with a 10-s exposure time and a variable gain setting (40–100) specific to each analyte.

**Figure 1:**
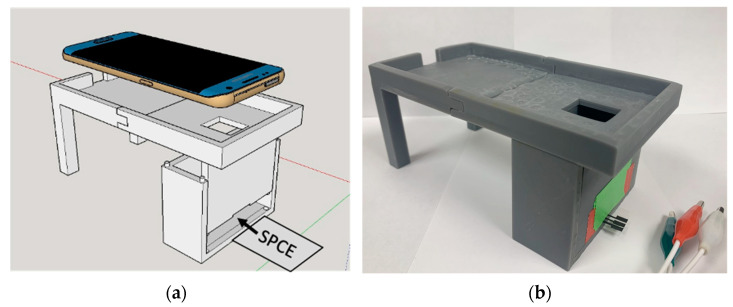
SketchUp 2017 design (**a**) and photograph (**b**) of the light-tight housing and phone holder. The optical detector rests in the tray and uses the small window to image the light-tight chamber. The SPCE is inserted into the closed chamber through a narrow slit as shown. The design is modular and the phone holder can be swapped out with the CCD camera holder (not shown).

*Fabrication of Light-Tight Housing*. The free version of Sketchup 2017 was used to design a lightproof housing for a SPCE beneath a holding tray for a mobile phone or CCD. An expanded schematic for the housing and a model of the Samsung Galaxy S7 Edge mobile phone used in this work is adjacent to a photograph of the housing (Figure 1). When in use, the lower chamber of the housing slides open so that a SPCE can be taped to the chamber’s bottom surface, and slides closed so that one end of the electrodes will extend out of the chamber while the working surface remains inside the dark environment. The potentiostat leads (alligator clips) are then attached to the exposed ends of the SPCE. A small window was placed in the top of the chamber so that the mobile phone or CCD camera could image the electrode without letting in ambient light. After taking photographs at different distances (Figure S4), the chamber was designed with a height of 6 cm so that the cameras could be as close to the electrode surface as possible while still being able to achieve a clear focus on the electrode. Once designed, the light-tight housing was printed using a FormLabs Form 2 printer with opaque gray resin. The resin was allowed to dry for 24 h after printing before the housing was assembled and used.

*Typical ECL Analysis Procedure*. It was first necessary to equilibrate each SPCE with ~5–8 trials. For example, a small aliquot of 2–3 drops of a solution containing 5.0 mM Ru(bpy)_3_^2+^ and 0.05 mM amine in buffer solution was deposited into the reservoir on the electrode surface. The solution was mixed gently using a plastic transfer pipette. Then, 8 separate 10-s applications of a 1.10 V potential were performed, using the transfer pipette to gently mix the DBAE solution in between each. The 2–3 drop aliquot was replaced after every 2 potential applications to ensure that its DBAE content was not depleted. The solution was then withdrawn and the electrode surface gently wicked dry. The SPCE was then sealed within the light-tight housing and the chosen optical detector was affixed to cover the chamber window. The potentiostat was configured to deliver 10 s of fixed potential to the electrode and triggered simultaneously with the chosen optical detector. The aliquot of analyte solution was replaced after every trial. Optical measurements of ECL production were exported as .jpeg files to a lab computer. The freely available image analysis program ImageJ [47] was used to analyze the resulting images. The mean value of red pixels (1–255) on the surface of the working electrode was calculated as a proxy for the intensity of red-orange ECL given off by Ru(bpy)_3_^2+^. The ‘circular selection’ tool within ImageJ was used to ensure that a consistent region was analyzed in each image file.

*Milk Sample Preparation*. Skim milk samples were centrifuged in an Eppendorf Centrifuge 5430 at 13,000 rpm for 25 min to separate colloids and fats which may interfere with ECL production and occlude detection. The resulting supernatant (approximately one-half of the total volume) was removed and diluted fourfold into a solution containing 5.0 mM Ru(bpy)_3_^2+^ for analysis.

## 3. Results

### 3.1. Electrode Characterization via Cyclic Voltammetry

#### 3.1.1. Ferricyanide

The stencil-printed electrodes were fabricated so that the working, counter, and reference were each made from the carbon ink/graphite mixture (Figure S3). Cyclic voltammetry (CV) was utilized to investigate the effects of adding a layer of Ag paint to the reference electrode. Five sequential CV’s of the one-electron reduction of ferricyanide (Fe(CN)_6_^3−^, Equation (S1)) were collected at each electrode. Each CV was collected after a two-minute wait time to allow for diffusion layer equilibration. Figure S5C,D compare CV’s of ferricyanide solutions at stencil-printed electrodes with a carbon reference and an Ag reference, respectively. As a control, CV’s at the stencil-printed electrodes with a conventional Ag, AgCl reference electrode and CV’s at a glassy carbon (GC) working electrode with an Ag, AgCl reference electrode were also collected (Figure S5A,B). Each group of CV’s was collected at the same electrode and referred to as “intra-electrode” studies. All CV’s show two peaks: a cathodic wave for the reduction of ferricyanide to ferrocyanide on the forward scan and an anodic wave for the oxidation of the ferrocyanide back to ferricyanide on the reverse scan (Equation (S1)). Table S1 shows the mean half-wave potential (*E*_1/2_), peak separation (Δ*E_p_*) and peak current ratio (*i_pc_*/*i_pa_*) values for each set of CV’s. The *E*_1/2_ values vary as the reference electrode is changed, as expected. However, for each specific electrode, there is little drift and values remain constant with <5 mV changes for all except the carbon reference. The shape of each CV indicates a reversible electrochemical process at each electrode. The other electrochemical data extracted from the CV’s (Δ*E_p_* and *i_pc_*/*i_pa_*) obtained from the cyclic voltammograms tell about the reversibility of the reduction of ferricyanide at the SPE’s. Ideally, Δ*E_p_* is around 59/*n* mV (*n* = mol of electrons) and *i_pc_*/*i_pa_* is ~1 for a reversible process [48]. The CG electrode in the conventional electrochemical cell exhibits the most ideal behavior, as expected. The stencil-printed electrodes with Ag and Ag, AgCl references both have an *i_pc_*/*i_pa_* of ~1.2, similar to the conventional electrode, while the carbon reference has a ratio of ~1.3. The peak separation is much greater for the stencil-printed electrodes than the conventional electrode. This is most likely due to the greater resistance of the electrode material, as commercial carbon inks contain proprietary binder materials that are not conductive. [49,50] However, the use of the Ag reference gives sharper and more defined peaks than the carbon reference, which has broader and flatter peaks.

As the SPCE’s are hand-made, “inter-electrode” studies were also performed to investigate electrode-to-electrode reproducibility. The experiments in Figure S5 and Table S1 were repeated with two additional SPCE’s for each working/reference combination and the mean and pooled standard deviations were calculated for each set. Table S2 shows the *E*_1/2_, Δ*E_p_* and *i_pc_*/*i_pa_* values for the CV’s collected. There is good agreement in the *E*_1/2_ values within each type of reference electrode. Using an Ag or Ag, AgCl reference gives minimal drift (<10 mV) in *E*_1/2._ Peak separation values remained similar to the intra-electrode study with similar standard deviations, as did *i_pc_*/*i_pa_*. Using carbon as a reference was observed to increase the variability of the electrochemical data. As the Ag paint dries within a few minutes, we decided that this additional step was beneficial to obtain an improved electrochemical response on par with using a conventional reference electrode. Another advantage of the stencil-printed electrodes are the larger currents observed. This phenomenon is most likely due to the increased surface area of the printed electrodes over the GC and has been observed by others [43].

#### 3.1.2. Ru(bpy)_3_^2+^

**Figure 2:**
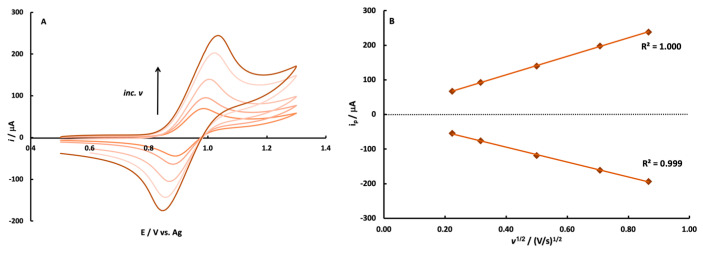
Cyclic voltammograms (**A**) and peak current versus the square root of scan rate (**B**) for a solution containing 5.0 mM Ru(bpy)_3_^2+^ in 0.10 M PBS (pH 7.4). Scan rates investigated were 0.05, 0.10, 0.25, 0.50, and 0.75 V/s.

The electrochemistry of Ru(bpy)_3_^2+^ oxidation was also investigated via cyclic voltammetry at an SPCE with an Ag reference, as the ECL reaction requires its one-electron oxidation (Figure S2, step 1). The CV data collected demonstrates that the oxidation of Ru(bpy)_3_^2+^ is reversible and diffusion-controlled at these electrodes. Figure 2 shows CV’s collected as a function of scan rate within the range of 0.05–0.75 V/s. The CV’s collected are characteristic of the one-electron oxidation and re-reduction of Ru(bpy)_3_^2+^. The peak current for both the anodic and cathodic waves is linear with the square root of scan rate, as described by the Randles–Sevcik equation, for a reversible diffusion-controlled redox reaction [51]. Visual inspection of the CV’s along with the additional representative CV data shown in Table S3 (Δ*E_p_* and *i_pa_*/*i_pc_*) demonstrate that the oxidation is reversible. The *E*_1/2_ value remains constant (~0.94 vs. Ag) with scan rate. The peak separation increased with the increasing scan rate, as might be expected for these electrodes which have some resistance from binder materials. The electrochemical investigation and reference electrode comparison deemed that a stencil-printed carbon electrode with an Ag quasi-reference electrode would be acceptable for generating Ru(bpy)_3_^2+^ ECL.

### 3.2. Electrochemistry of DBAE and Biogenic Amines with Ru(bpy)_3_^2+^

**Figure 3:**
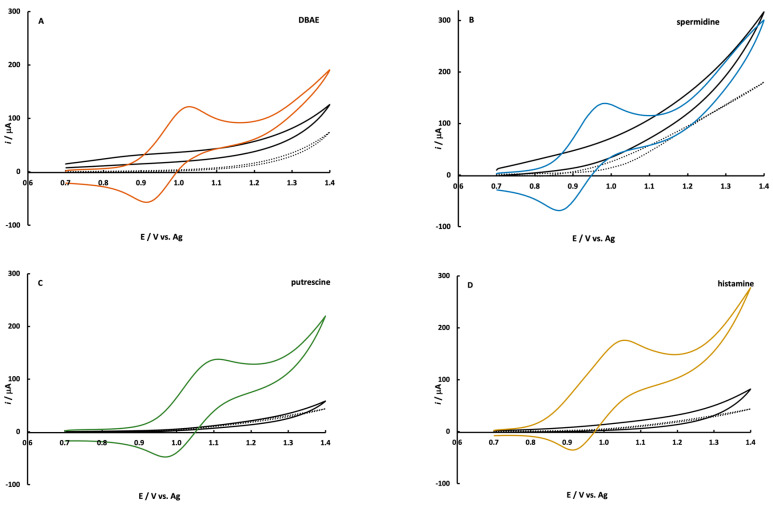
Cyclic voltammograms of 5.0 mM Ru(bpy)_3_^2+^ plus (**A**) 0.85 mM DBAE, (**B**) 1.35 mM spermidine both in pH 7.4 PBS; (**C**) 1.35 mM putrescine, and (**D**) 1.35 mM histamine both in pH 9.0 borate buffer. The black line corresponds to a 2.0 mM solution of each amine and the dotted line is the background scan. All CV’s were collected at a scan rate of 0.1 V/s.

The ECL co-reactant mechanism between amines and Ru(bpy)_3_^2+^ has been investigated with commonly used co-reactants such as tripropylamine [32,37] and 2-(dibutylamino)ethanol (DBAE) [35]. These studies can be generalized to an amine mechanism via the route shown in Figure S2. The mechanism that typically dominates when the Ru(bpy)_3_^2+^ concentration is high and the amine concentration is low is the catalytic mechanism (EC’) as shown by steps 1, 2a, then 3–5 in Figure S2. Under these conditions, Ru(bpy)_3_^2+^ is heterogeneously oxidized at the electrode while the primary route of amine oxidation is homogeneous (step 2a) [37,38]. The CV’s shown in Figure 3 also support the homogeneous oxidation of amine by Ru(bpy)_3_^2+^. It is well known that the heterogeneous oxidation of aliphatic amines in an aqueous solution is difficult due to kinetic limitations. The reaction requires the transfer of oxygen from water and the most common electrode materials cannot support this mechanism [21,52,53,54]. The CV’s of amine solutions at SPCE’s in Figure 3 demonstrate a little-to-no oxidation current above background scans. DBAE and spermidine exhibit a small oxidation wave above the background with no discernable peak-shape in their CV’s. When CV’s are collected for solutions containing both amine and Ru(bpy)_3_^2+^, the characteristic Ru(bpy)_3_^2+^ CV is observed, as shown in Figure 3, with little contribution from the amine [38].

This electrochemical investigation demonstrated that these electrodes would be viable for generating Ru(bpy)_3_^2+^ ECL. The subsequent experiments demonstrate the optimization of the system and determination of analytical figures of merit. Prior work in our lab and literature searches [23,24,25,26,27,28,55] indicated that a Ru(bpy)_3_^2+^ concentration of ~4–5 mM would lead to the highest signal-to-background ratio. Prior literature reports have demonstrated that the ECL reaction between Ru(bpy)_3_^2+^ and amines provides the highest signal at neutral to slightly basic pH values [32,33,34,35,55]. For work with biogenic amines, the general consensus is that pH 7–9 leads to the highest S/N, but pH 9 can be preferable for primary amines such as putrescine and histamine [23,24,25,26,27,28]. Therefore, in the next section, ECL work was performed at the optimal pH for each amine. However, we found that the signals were sufficiently high for the secondary amine spermidine, and that either pH (7 or 9) was sufficient.

### 3.3. Verification of Light-Tight Housing Improvements

**Figure 4:**
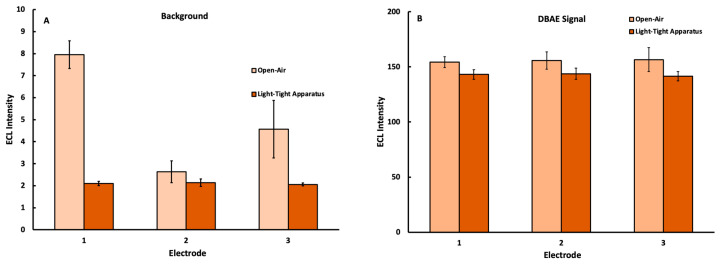
ECL signal collected with a mobile phone detector under an applied potential of 1.1 V for three different electrodes using solutions containing 5.0 mM Ru(bpy)_3_^2+^ plus (**A**) 0.00 mM and (**B**) 0.45 mM DBAE with trials captured either in open air or using the light-tight housing in Figure 1 (n = 10 at each electrode).

The ECL production of the well-known Ru(bpy)_3_^2+^ DBAE reaction system [35] was used as a benchmark to quantify the improvements to precision afforded by the implementation of the 3D-printed light-tight housing. Figure 4 shows the elimination of ambient light from the instrument background provided a lower and more consistent noise level when measured within the light-tight housing. Here, the inter-electrode variability was reduced due to the consistently dark environment within the housing. This, in turn, allowed for a more precise measurement of and correction for the mobile phone instrument background in subsequent trials.

### 3.4. Optimization of Potential

**Figure 5:**
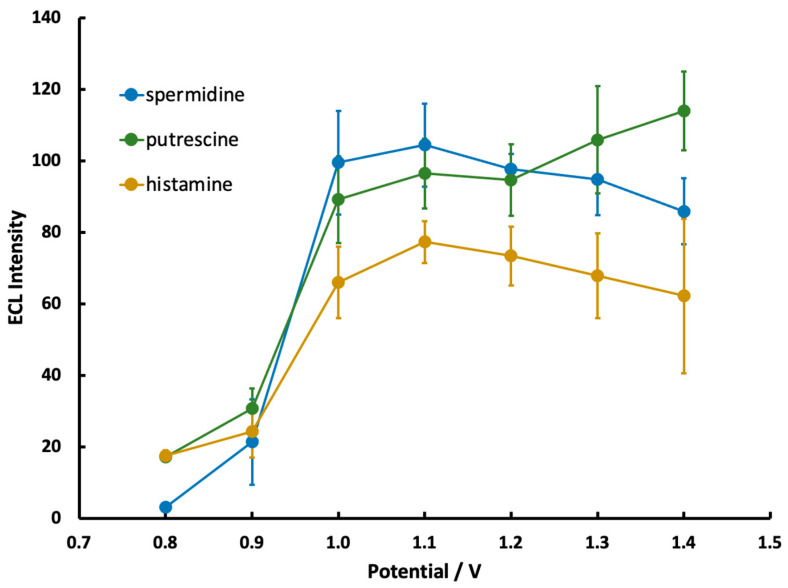
ECL intensity from the mobile phone detector as a function of applied potential for solutions containing 5.0 mM Ru(bpy)_3_^2+^ and 1.35 mM spermidine, putrescine, or histamine (n = 6 over two different electrodes).

The effect of applied potential on ECL production is shown in Figure 5 for spermidine, putrescine, and histamine. Trials at each potential were split over two electrodes and then averaged to minimize the possible effects of individual electrode fluctuations. Little ECL production was observed at potentials <1.0 V due to insufficient Ru(bpy)_3_^2+^ oxidization, which correlates with the CV data in Figure 3. At potentials >1.3 V, ECL production generally decreased or became more variable as additional non-analyte species, such as water or buffer, started to oxidize. The production of O_2_ in particular creates bubbles on the electrode surface that result in a patchy and inconsistent appearance of the ECL glow.

Based on the above constraints, applied potentials of 1.0–1.2 V were identified as areas of optimal ECL production for spermidine and putrescine, while a region of 1.1–1.2 V was identified for histamine. A potential within the above ranges was selected for subsequent trials of each amine.

### 3.5. Comparison of Mobile Phone and CCD Detector Linearity

**Figure 6:**
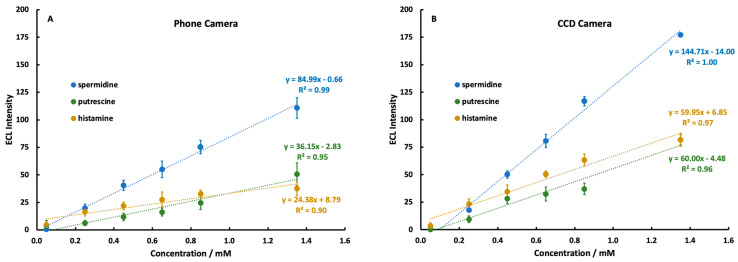
Background-subtracted calibration curves for 0.05–1.35 mM spermidine, putrescine, and histamine added to 5.0 mM Ru(bpy)_3_^2+^, detected by a Samsung Galaxy S7 Edge phone camera (**A**) and a CCD Camera (**B**). Each curve is the average of 3 separate studies on independent electrodes, and each study was n = 5 at every concentration.

To assess the quality of a mobile phone camera as an optical detector for ECL in biogenic amines, identical calibration curves were prepared for each amine solution and measured with both detectors (Figure 6). Based on the results of the potential optimization study, a potential of 1.10 V was used for spermidine and putrescine, while a potential of 1.2 V was used for histamine. Solutions containing spermidine were prepared initially in a pH 7.40 0.10 M phosphate-buffered solution, since previous studies [25,28] had demonstrated good ECL production by spermidine around pH 7.0. However, tests conducted after the calibration curve experiments showed little difference in spermidine ECL production in a pH 9.00 solution. Our recommendation would be to prepare all solutions in a pH 9.00 buffer for simplicity.

A direct comparison of the slopes in Figure 6 reveals a decrease in sensitivity by around a factor of two in the mobile phone compared to the CCD. Despite this, linearity was achieved in the signal response of each amine using both detection methods. The mobile phone detection system suffered expected decreases in performance compared to the CCD camera, but still functioned as an analytically capable tool, particularly for spermidine. Spermidine displayed a markedly higher ECL response than putrescine or histamine due to the presence of a secondary amine group in its structure.

Oral toxicity levels for the biogenic amines detected here have been proposed to be 500 ppm for histamine, 2000 ppm for putrescine, and 600 ppm for spermidine [12]. The limits of detection reported here (Table 1) are well below these levels, and therefore, this technique could make measurements on samples that have been diluted by the addition of the Ru(bpy)_3_^2+^ solution. Therefore, the potential exists for the system to make safety-relevant assessments about the presence or absence of histamine and other biogenic amines in a food product.

### 3.6. Comparison of Mobile Phone and CCD Detector Precision

Table 1 shows a direct comparison of measurement repeatability (n = 10) of ECL for the detection of 0.45 mM of each biogenic amine with either the mobile phone or CCD as a detector. Applied potentials were the same as those used for the calibration curves. The mobile phone camera was slightly more variable, though it marginally outperformed the CCD camera in the study of putrescine. Relative standard deviation values for both detection methods overlapped, indicating that in repeatability, the detection methods are moderately comparable.

### 3.7. Interference Study

**Figure 7:**
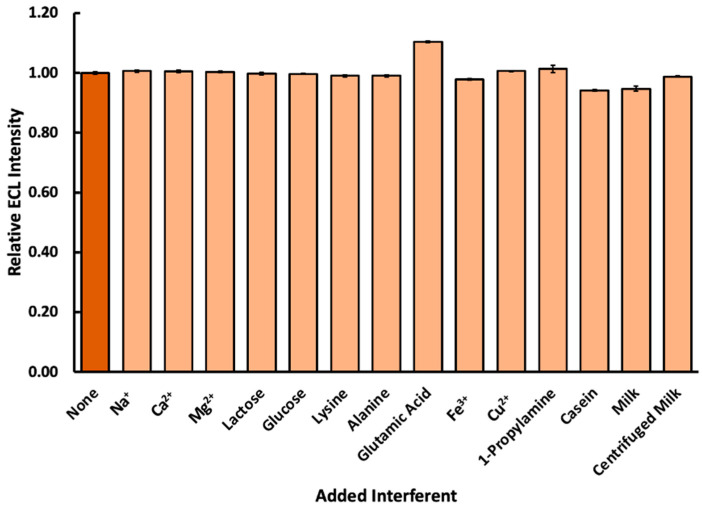
Effect of 1.0 mM interfering species on ECL production at 1.10 V when added to a solution containing 1.0 mM spermidine and 5.0 mM Ru(bpy)_3_^2+^ (n = 4).

Real food samples present complex matrices in which a number of possible chemical interferences may exist. Before the Ru(bpy)_3_^2+^ ECL reaction system could be measured in a real-world sample, it was necessary to confirm that a representative selection of common biological molecules and ions would not interfere in ECL production from biogenic amines. Figure 7 shows a comparison of a 1.0 mM spermidine and 5.0 mM Ru(bpy)_3_^2+^ ECL reaction at 1.10 V with the presence or absence of 1 mM interferents that could be found in a dairy sample such as milk.

Most ions and small biomolecules produced no meaningful interference of ECL production. Glutamic acid led to a partial increase in ECL signal, likely because the deprotonated carboxylic acid functional group may have been able to participate in the radical formation and transfer steps of the ECL mechanism. Fortunately, the concentration of free glutamic acid in real bovine milk samples has been documented as being much lower than the 1.0 mM tested here and is typically in the regime of 0.2 mM [56]. Nonetheless, multiple amino acids are likely to be present and create a stable ECL background in certain types of biological samples; thus, the decision was made to select a multiple standard addition analysis for the determination of biogenic amines. Additionally, the presence of 1.0 mM casein mildly inhibited ECL production. This may be because casein forms colloids in aqueous solutions—structures which trap and sequester the biogenic amines. The presence of this interference informs the decision to centrifuge milk samples before testing, as colloids may be removed from dairy products with centrifugation.

### 3.8. Application to Milk Samples

The capability of a mobile phone detector for measuring biogenic amine ECL with Ru(bpy)_3_^2+^ was tested practically by constructing a multiple standard addition curve of spermidine to prepared skim milk samples, with the goal of making a test measurement of the existing amine content in unexpired milk. Figure S6 shows the resulting curve when milk samples were spiked with 0–0.50 mM spermidine and ECL production was measured at 1.10 V with either a mobile phone camera or a CCD camera. As seen by an inspection of the x-intercepts of the graphs, the two methods agreed well with one another, with an intercept of 0.30 mM with the mobile phone detection and 0.28 mM using the CCD detection (n = 3 at each concentration). These values correlate with the 350 and 320 ppm original concentration, which is reasonable for a total amine concentration in a milk or dairy sample [12,57,58]. Note that these electrodes, although free from the interferences in Figure 7, would not be able to discriminate against different BA without coupling to a separation method. This experiment demonstrates the proof of concept and sample preparation and coupling to a separation method will be the subjects of future studies.

## 4. Discussion

As previously mentioned, toxicity levels for various BA have been proposed and can vary depending on the amine and the type of food product. Histamine receives the most attention from groups such as the European Food Safety Authority (EFSA), Food and Drug Administration, Food Safety Commission of Japan (FSCJ), and the World Health Organization (WHO). However, other BA can cause illness on their own or enhance histamine’s toxicity. Depending on the study or food product, measurements may need to be made on maximum tolerable limits of specific BA [2,3,10,12,15] or total BA [2,5,12]. The electrodes reported here would be able to measure a total amine content or, when coupled to a separation method, measure the concentration of individual BA.

The electrodes, along with ECL detection using a mobile phone encased in an inexpensive 3D-printed light-tight casing, demonstrate the potential of a portable and inexpensive ECL detection method for BA. In this work, we verified and optimized the voltammetric response of the electrodes. We also verified the use of a light-tight casing for the measurement, while optimizing the detection voltage and solution conditions. This work also compared the analytical figures of merit of the phone camera to a lab-grade CCD camera using Ru(bpy)_3_^2+^ as the emitter. The CCD camera narrowly outperformed the mobile phone, demonstrating the viability of the phone camera for ECL detection. Finally, this work demonstrated that the ECL method, while unable to differentiate between various BA without a separation method, had a response free from many other interferences found in a dairy sample such as milk. Future investigations could investigate other ECL emitters such as luminol [59,60,61,62] or various nanomaterials [36,63].

## 5. Conclusions

The verification of a mobile phone’s capability for analytical optical measurements is a positive step forward within the context of developing an accessible and low-cost set of instrumentation for ECL analysis. Despite an expected shortcoming in sensitivity, the mobile phone’s quantitation of ECL from Ru(bpy)_3_^2+^ and biogenic amines was comparable in precision and linear range to that of a CCD camera. Even more importantly, the mobile phone detection of ECL in a real-world milk sample produced a determination of spermidine content on par with a CCD camera.

## Figures and Tables

**Table 1:** Mobile Phone and CCD Limits of Detection, Linear Range and Precision for Biogenic Amine ECL.

Analyte			Mobile Phone		CCD Camera	
	LOD μM (ppm) ^1^	Linear Range μM (ppm) ^1^	Precision %RSD^2^	LOD μM (ppm) ^1^	Linear Range μM (ppm) ^1^	Precision %RSD ^2^
Spermine	127 (18)	50–1350 (7–196)	7.5	39 (5.7)	50–1350 (7–196)	2.6
Putrescine	425 (38)	50–1350 (4–120)	5.6	149 (13)	50–1350 (4–120)	7.6
Histamine	421 (47)	50–1350 (6–150)	9.9	228 (25)	50–1350 (6–150)	5.1

^1^ Concentrations in ( ) are parts-per-million. ^2^ Precision reported as % RSD (n = 10).

## Data Availability

The data that support the findings of this study are available on request from the corresponding author. The data are not publicly available due to privacy or ethical restrictions.

## Supplementary Materials

The following supporting information can be downloaded at: https://www.mdpi.com/article/10.3390/s22187008/s1, Figure S1: Structures of (A) tris(2,2′-bipyridyl)ruthenium(II) and (B) amines investigated in this work. Figure S2: Mechanism of oxidative-reductive ECL mechanism of amines with Ru(bpy)_3_^2+^. Figure S3: Stencil printing carbon paste through a laser-cut transparency stencil. Figure S4: Photographs taken of a 3.0 mm diameter SPCE generating ECL. Figure S5: Intra-electrode cyclic voltammograms of 4.0 mM ferricyanide in 1.0 M KNO_3_ at carbon electrodes. Figure S6: Multiple standard addition graphs. Table S1: Intra-electrode CV data for the reduction of ferricyanide. Table S2: Inter-electrode CV data for the reduction of ferricyanide. Table S3: Electrochemical data for the oxidation of Ru(bpy)_3_^2+^ at stencil-printed electrodes.
