# Supplementary material for: Comparison of Mobile Phone and CCD Cameras for Electrochemiluminescent Detection of Biogenic Amines
Source: Sensors (Basel). 2022 Sep 16;22(18):7008. doi: 10.3390/s22187008 (PMC9503902; doi:10.3390/s22187008)
Supplement: Supplementary file 1 [file sensors-22-07008-s001.zip › sensors-1891156-supplementary.pdf]

Supplemental Material for Comparison of Mobile Phone and CCD Cameras for Electrochemiluminescent Detection of Biogenic Amines

Nic Heckenlaible [1], Sarah Snyder [1], Patrick Herchenbach[1], Alyssa Kava [2], Charles S. Henry [2], and Erin M. Gross [1,*]

[1]Department of Chemistry and Biochemistry, Creighton University, Omaha, NE, USA
[2]Department of Chemistry, Colorado State University, Fort Collins, CO, USA

[*]Corresponding Author E-mail Address eringross@creighton.edu

Chemical structures and ECL mechanism

The chemical species 2-(dibutylamino)ethanol (DBAE) is a well-studied exemplary ECL co-reactant [1]. The mechanism for the oxidation-reduction ECL production between alkyl amines and $Ru(bpy)_3^{2+}$ is shown in in Figure S2 [2-3]. The general scheme of the ECL mechanism involves the oxidation of both $Ru(bpy)_3^{2+}$ and co-reactant, after which the amine rearranges to form a free radical. The radical reduces $Ru(bpy)_3^{3+}$ in a process that creates an electronically excited $*Ru(bpy)_3^{2+}$ state. Finally, $*Ru(bpy)_3^{2+}$ relaxes to $Ru(bpy)_3^{2+}$ and emits a photon with a characteristic wavelength of 620nm.

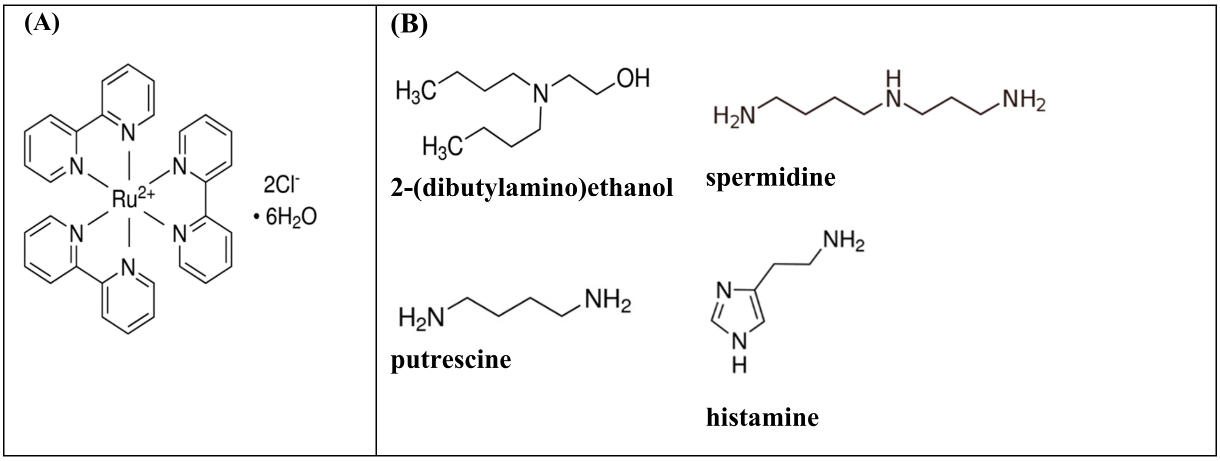

Figure S1. Structures of (A) tris(2,2'-bipyridyl)ruthenium(II) and (B) amines investigated in this work.

It has been noted that ECL mechanisms involving amines work most efficiently with tertiary amines [4], though secondary and primary amines may also be substituted with only a moderate decline in signal response. One such class of primary, secondary, and tertiary amines to which ECL studies have been applied are the biogenic amines.

$$(1)\quad Ru(bpy)_3^{2+} \rightarrow Ru(bpy)_3^{3+} + e^-$$

$$(2a)\quad Ru(bpy)_3^{3+} + R_2NH \rightarrow Ru(bpy)_3^{2+} + R_2NH^{+\cdot}$$

$$(2b)\quad R_2NH \xrightarrow{-e^-} R_2NH^{+\cdot}$$

$$(3)\quad R_2NH^{+\cdot} \xrightarrow{-H^+} R^{\cdot}$$

$$(4)\quad R^{\cdot} + Ru(bpy)_3^{3+} \rightarrow P + {}^*Ru(bpy)_3^{2+}$$

$$(5)\quad {}^*Ru(bpy)_3^{2+} \rightarrow Ru(bpy)_3^{2+} + h\nu\,(\sim 620\ nm)$$

Figure S2. Mechanism of oxidative-reductive ECL mechanism of amines with $Ru(bpy)_3^{2+}$. $R^{\cdot}$ represents the strongly reducing free radical and P represents a product.

<u>Electrode Fabrication</u>

Stencil-printed carbon electrodes were fabricated as described in the experimental section [5]. with photographs of the process and the end product shown in Figure S3.

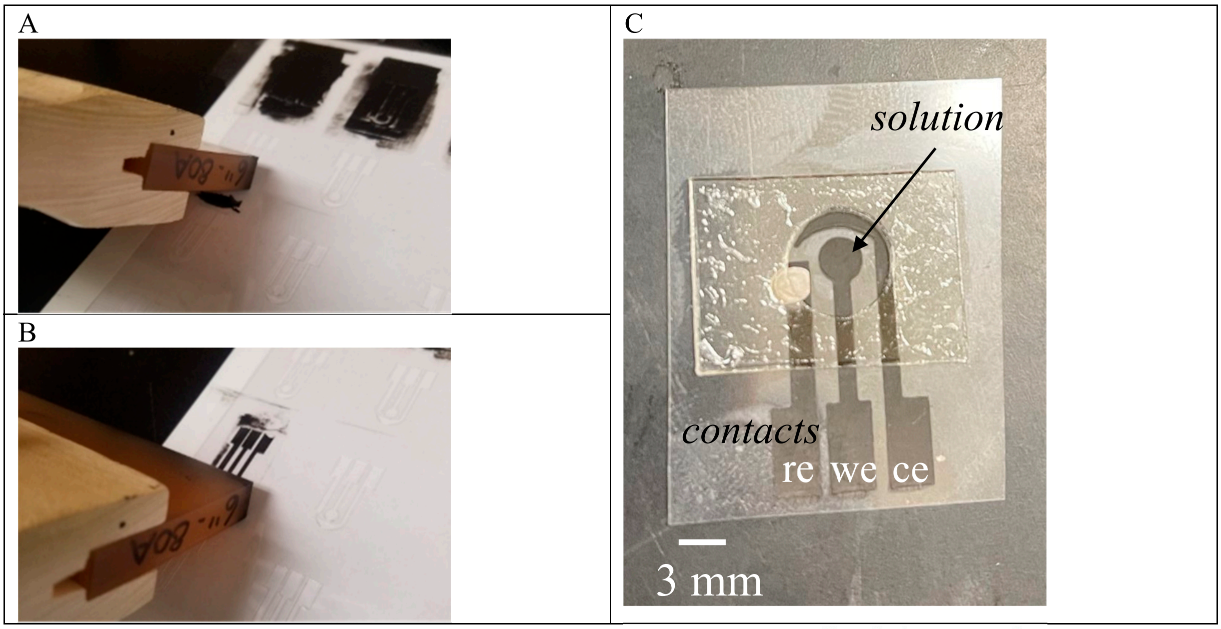

Figure S3. Stencil printing carbon paste through a laser-cut transparency stencil and (A-B) completed SPCE with reservoir attached (C). A few drops of solution are placed in the reservoir and contact is made to the reference (re), working (we) and counter (ce) electrodes via the contact pads at the bottom.

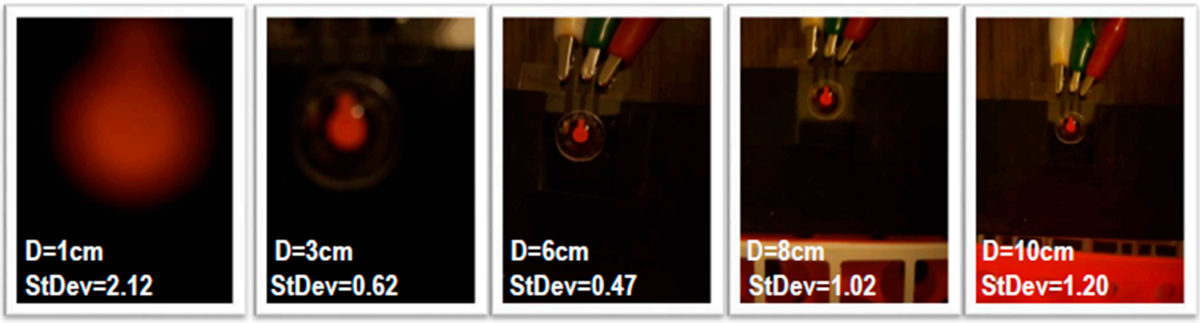

Figure S4. Photographs taken of a 3.0 mm diameter SPCE generating ECL from a solution of 5.0 mM $Ru(bpy)_3^{2+}$ and 0.050 mM DBAE under an applied potential of 1.10. Standard deviations were calculated from the image analysis of red pixel mean on ImageJ (n = 5).

Cyclic voltammetry at stencil-printed electrodes

These electrodes are fabricated so that the working, counter and reference are each made from the carbon ink/graphite mixture. We investigated the effects of painting the reference with Ag paint on the stability of the cyclic voltammetry. Here, we looked at the CV's of the one-electron reduction of ferricyanide ($Fe(CN)_6^{3-}$). For each reference electrode, five CV's were collected, with a two minute wait time in between. The data table shows the mean and standard deviations of the peak cathodic and anodic currents.

$$Fe(CN)_6^{3-} + e^- \Leftrightarrow Fe(CN)_6^{4-} \quad \text{(Equation S1)}$$

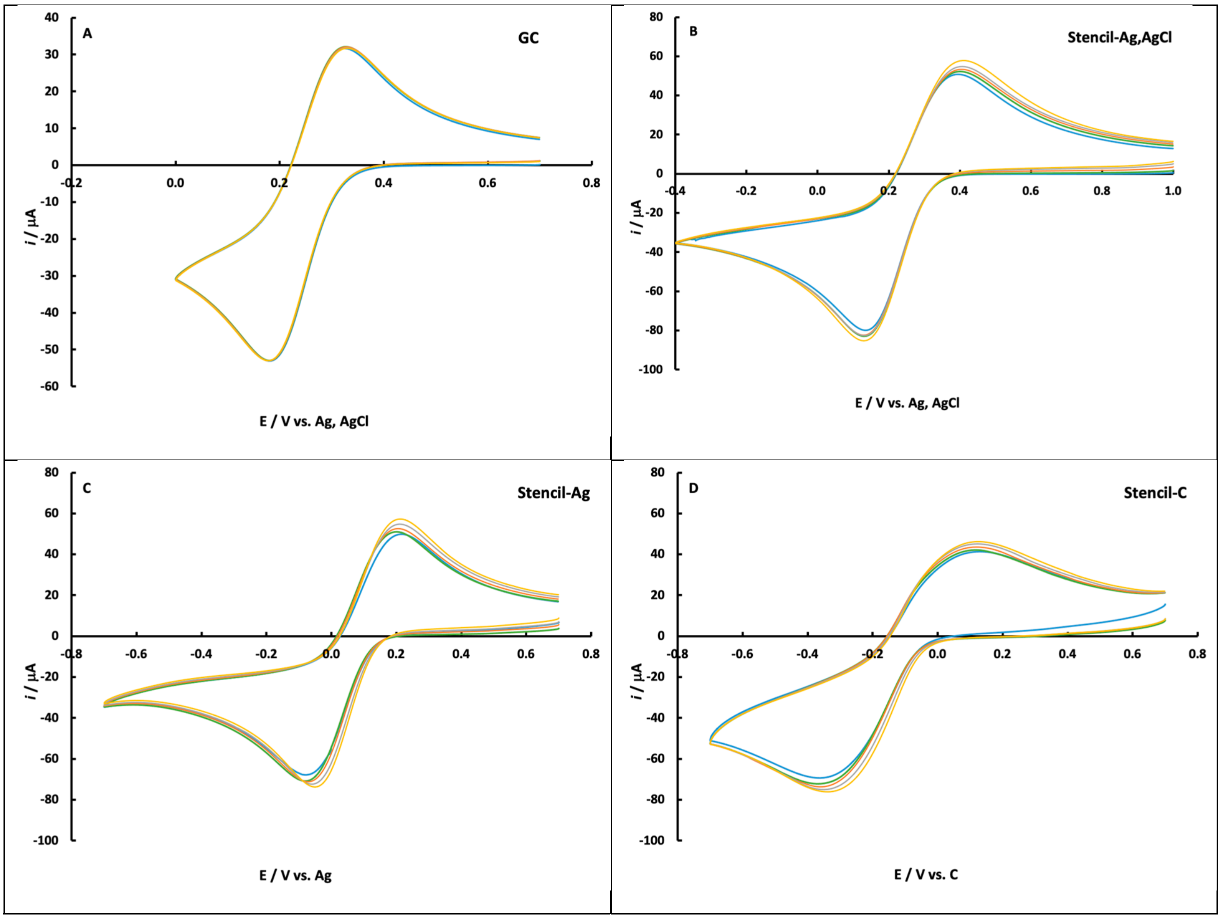

Figure S5. Intra-electrode cyclic voltammograms of 4.0 mM ferricyanide in 1.0 M KNO_3 at carbon electrodes (v = 0.10 V/s). A) glassy carbon with Ag, AgCl reference and Pt counter electrode. Stencil-printed carbon with B) Ag, AgCl reference C) Ag paint reference and C) carbon reference. All stencil printed electrodes have a carbon counter electrode.

Table S1: Intra-electrode CV data for the reduction of ferricyanide*

Working	Reference	$E_{1/2}$ (V)	ΔE_p (mV)	i_{pc}/i_{pa}
**Stencil**	**Ag, AgCl**	0.268 ± 0.002	272 ± 8	1.22 ± 0.01
**Stencil**	**Ag**	0.110 ± 0.005	305 ± 16	1.16 ± 0.02
**Stencil**	**Carbon**	-0.183 ± 0.050	308 ± 54	1.31 ± 0.11
**GC**	**Ag, AgCl**	0.254 ± 0.001	110 ± 4	1.21 ± 0.01

*Mean ± standard deviation (n = 5 for each electrode)

Table S2: Inter-electrode CV data for the reduction of ferricyanide *

Working	Reference	$E_{1/2}$ (V)	ΔE_p (mV)	i_{pc}/i_{pa}
**Stencil**	**Ag, AgCl**	0.269 ± 0.003	350 ± 7	1.31 ± 0.04
**Stencil**	**Ag**	0.101 ± 0.008	289 ± 21	1.13 ± 0.05
**Stencil**	**Carbon**	-0.151 ± 0.030	384 ± 34	1.61 ± 0.09
**GC**	**Ag, AgCl**	NA	NA	NA

*Mean ± pooled standard deviation (3 electrodes, n = 5 for each electrode)

Cyclic Voltammetry of $Ru(bpy)_3^{2+}$

Table S3: Electrochemical data for the oxidation of $Ru(bpy)_3^{2+}$ at stencil-printed electrodes

Scan rate (V/s)	$E_{1/2}$ (V) vs. Ag	ΔE_p (mV)	i_{pa}/i_{pc}
0.05	0.938	96	1.22
0.10	0.938	111	1.22
0.25	0.937	136	1.18
0.50	0.939	166	1.23
0.75	0.941	187	1.23

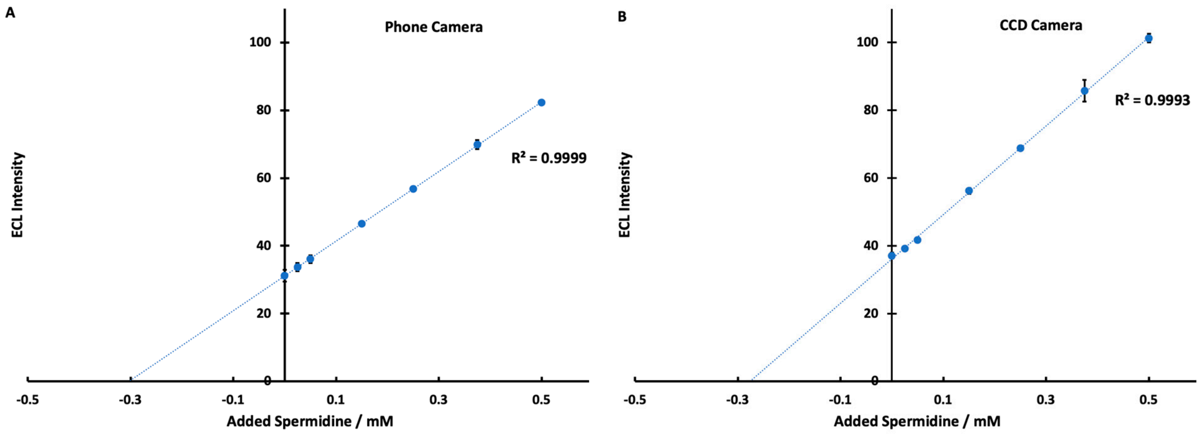

Figure S6. Multiple standard addition of 0-0.50 mM spermidine and 5.0 mM $Ru(bpy)_3^{2+}$ to 4x diluted centrifuged skim milk samples captured at 1.10 V with a (A) mobile phone camera and (B) CCD camera.

References

1. Liu, X.; Shi, L.; Niu, W.; Li, H. and Xu, G. Environmentally Friendly and Highly Sensitive Ruthenium(II) Tris(2,2′-bipyridyl) Electrochemiluminescent System Using 2-(Dibutylamino)ethanol as Co-Reactant. *Angew. Chem. Int. Ed.* **2007**, *46*, 421-424. DOI: 10.1002/anie.200603491
2. Miao, W.; Choi, J.-P. and Bard, A.J. Electrogenerated Chemiluminescence 69: The Tris(2,2‘-bipyridine)ruthenium(II), (Ru(bpy)32+)/Tri-n-propylamine (TPrA) System Revisited-A New Route Involving TPrA•+ Cation Radicals. *J. Am. Chem. Soc.* **2002**, *124*, 14478-14485. DOI:10.1021/ja027532v
3. Hu, L.; Xu, G. Applications and trends in electrochemiluminescence. *Chem. Soc. Rev.* **2010**, *39*, 3275–3304. https://doi.org/10.1039/B923679C.
4. Knight, A.W. and Greenway, G.M. Relationship between structural attributes and observed electrogenerated chemiluminescence (ECL) activity of tertiary amines as potential analytes for the tris(2,2-bipyridine)ruthenium(II) ECL reaction. A review. *Analyst*, **1996**, *121*, 101R-106R. DOI:10.1039/AN996210101R
5. Martín-Yerga, D.; Álvarez-Martos, I.; Blanco-López, M.C.; Henry, C.S. and Fernández-Abedul, M.T. Point-of-Need simultaneous electrochemical detection of lead and cadmium using low-cost stencil-printed transparency electrodes. *Anal. Chim. Acta* **2017**, *981*, 24-33. DOI:10.1016/j.aca.2017.05.027
6. Heineman, W.R. and Kissinger, P.T. *Laboratory Techniques in Electroanalytical Chemistry, 2nd Ed.* Kissinger, P.T. and Heineman, W.R., Eds.; Marcel Dekker: New York, USA, 1996, pp 81-82.
7. Kissinger, P.T. and Heineman, W.R. Cyclic Voltammetry. *J. Chem. Ed.* **1983**, *60*, 702-706. DOI:10.1021/acs.jchemed.7b00361
